# Nanog induces suppression of senescence through downregulation of p27^KIP1^ expression

**DOI:** 10.1242/jcs.167932

**Published:** 2016-03-01

**Authors:** Bernhard Münst, Marc Christian Thier, Dirk Winnemöller, Martina Helfen, Rajkumar P. Thummer, Frank Edenhofer

**Affiliations:** 1 Stem Cell Engineering Group, Institute of Reconstructive Neurobiology, University of Bonn – Life & Brain Center and Hertie Foundation, Sigmund-Freud Str. 25, Bonn 53127, Germany; 2 Department of Biosciences and Bioengineering, Indian Institute of Technology Guwahati, Guwahati 781039, Assam, India; 3 Stem Cell and Regenerative Medicine Group, Institute of Anatomy and Cell Biology, Julius-Maximilians-University Würzburg, Koellikerstrasse 6, Würzburg 97070, Germany; 4 Department of Genomics, Stem Cell Biology & Regenerative Medicine, Institute of Molecular Biology, Leopold-Franzens-University Innsbruck, Technikerstraße 25, Innsbruck 6020, Austria

**Keywords:** Embryonic stem cell, Protein transduction, Pluripotency, Senescence, Cell reprogramming, p27^KIP1^

## Abstract

A comprehensive analysis of the molecular network of cellular factors establishing and maintaining pluripotency as well as self renewal of pluripotent stem cells is key for further progress in understanding basic stem cell biology. Nanog is necessary for the natural induction of pluripotency in early mammalian development but dispensable for both its maintenance and its artificial induction. To gain further insight into the molecular activity of Nanog, we analyzed the outcomes of Nanog gain-of-function in various cell models employing a recently developed biologically active recombinant cell-permeant protein, Nanog-TAT. We found that Nanog enhances the proliferation of both NIH 3T3 and primary fibroblast cells. Nanog transduction into primary fibroblasts results in suppression of senescence-associated β-galactosidase activity. Investigation of cell cycle factors revealed that transient activation of Nanog correlates with consistent downregulation of the cell cycle inhibitor p27^KIP1^ (also known as CDKN1B). By performing chromatin immunoprecipitation analysis, we confirmed bona fide Nanog-binding sites upstream of the p27^KIP1^ gene, establishing a direct link between physical occupancy and functional regulation. Our data demonstrates that Nanog enhances proliferation of fibroblasts through transcriptional regulation of cell cycle inhibitor p27 gene.

## Abstract (precis)

**Summary:** Nanog blocks cellular senescence of fibroblasts through transcriptional regulation of cell cycle inhibitor p27^KIP1^.

## INTRODUCTION

Pluripotent stem cells, such as embryonic stem cells (ESCs) and induced pluripotent stem cells (iPSCs), have tremendous potential in developmental biology as well as regenerative medicine due to their unlimited self renewal and unrestricted differentiation capacity. A thorough understanding of the molecular network of cellular factors, extracellular and intracellular signaling pathways, cell cycle regulation and microenvironment that establish and maintain self-renewal and pluripotency is key for the development of biomedical applications of stem cells (Boyer et al., 2005; Cox et al., 2011; Loh et al., 2006; Lowry and Quan, 2010). Establishment and maintenance of stem cell identity, particularly pluripotency, is regulated by a core network of transcription factors. Oct4 (also known as POU5F1), Sox2 and Nanog belong to this transcriptional circuit, and they play a pivotal role in self-renewal and maintenance of pluripotency (Chen et al., 2008; Chambers and Tomlinson, 2009; He et al., 2009; Pauklin et al., 2011). In concert with Oct4 and Sox2, Nanog governs pluripotent features in mouse and human cells (Boyer et al., 2005; Loh et al., 2006) by occupying the promoters of active genes encoding transcription factors, signal transduction components and chromatin-modifying enzymes. Expression of Oct4 and Sox2 is relatively homogeneous in pluripotent cells whereas Nanog exhibits a heterogeneous expression (Singh et al., 2007), with cells having elevated levels of Nanog exhibiting efficient self-renewal.

The homeodomain transcription factor Nanog is expressed during early embryonic development, in the inner cell mass, in ESCs and in the developing germline in mammals (Chambers et al., 2003, 2007; Mitsui et al., 2003). It has been shown that ESCs are sensitive to the dosage of Nanog. Overexpression of Nanog is sufficient to prevent differentiation in ESCs in absence of feeders and vital extracellular growth factors (Chambers et al., 2003; Darr et al., 2006; Mitsui et al., 2003). Downregulation of Nanog results in loss of pluripotency, reduction in cell proliferation and differentiation towards extraembryonic lineages (Chambers et al., 2003; Hyslop et al., 2005; Ivanova et al., 2006; Mitsui et al., 2003; Zaehres et al., 2005). Additionally, cell fusion experiments demonstrate that Nanog promotes the formation of pluripotent hybrids because Nanog stimulates pluripotent gene activation in neural stem cells, thymocytes and fibroblasts in a dose-dependent manner (Silva et al., 2006). Knockout studies have revealed that Nanog is dispensable for the housekeeping machinery of pluripotency given that the conditional deletion of Nanog in ESCs does, unexpectedly, not result in loss of pluripotency (Chambers et al., 2007). During development *in vivo*, pluripotency is not established without Nanog and inner cell mass cells are trapped in an intermediate stage (Silva et al., 2009), assigning Nanog an essential role for the natural acquisition, but not the maintenance, of pluripotency (Pan and Thomson, 2007; Saunders et al., 2013). Thus, a well-defined role of Nanog in self-renewal and the natural induction of pluripotency at the molecular level remains to be investigated.

Inducible gain-of-function systems permitting a precise control over the timing and dosage of gene product expression allow straightforward studies of stemness and cell reprogramming pathways at the molecular level. Using protein transduction, we generated cell-permeant versions of the core pluripotency factors Oct4 and Sox2 proteins (Bosnali and Edenhofer, 2008) to generate iPSCs (Thier et al., 2010, 2012a) and induced neural stem (iNS) cells (Thier et al., 2012b). Recently, we reported on a cell-permeant version of Nanog, and demonstrated that it promotes ESC proliferation and self-renewal in the absence of leukemia inhibitory factor by inhibiting endodermal specification in a Stat3-independent manner (Peitz et al., 2014). Here, we set out to study the function of Nanog in somatic cells as a means to analyze its contribution to self-renewal in cells in general and ESCs in particular. We show that biologically active cell-permeant Nanog induces enhanced proliferation in fibroblasts. Moreover, transient activation of Nanog consistently correlates with downregulation of cell cycle kinase inhibitor p27^KIP1^ (also known as CDKN1B). In addition, chromatin immunoprecipitation (ChIP) analysis reveals two distinct putative Nanog-binding sites upstream of the p27^KIP1^ gene. In conclusion, our data suggests that Nanog is a potential regulator of the p27^KIP1^ gene and enhances the proliferation of fibroblasts.

## RESULTS

### Nanog enhances the proliferation of fibroblasts and induces anchorage-independent growth

**Fig. 1.**
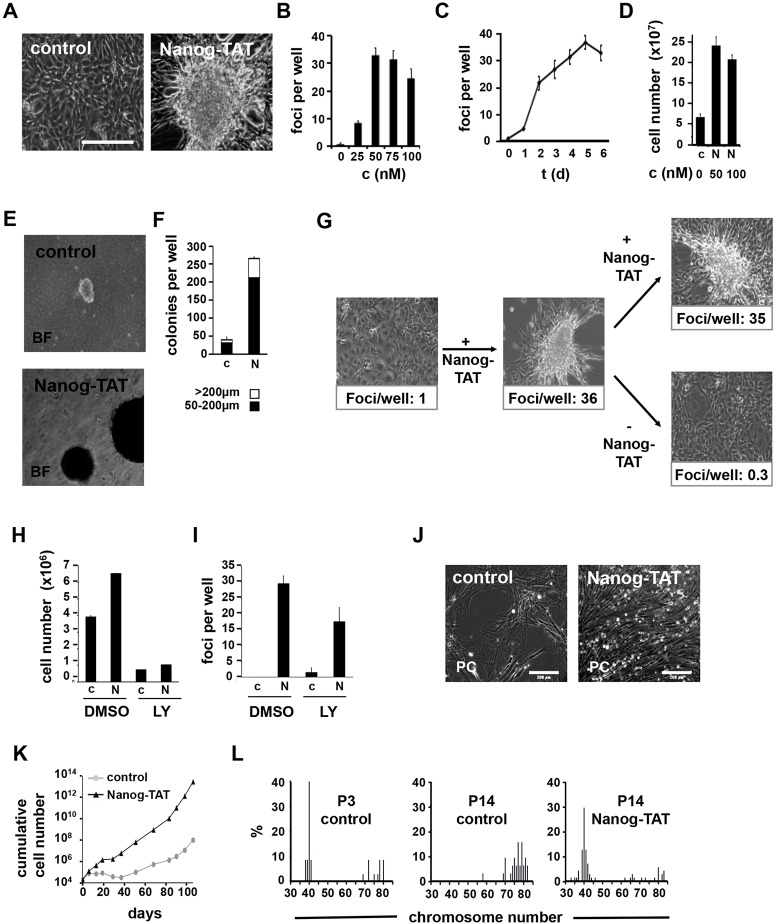
**Nanog-TAT induces anchorage-independent growth of NIH 3T3 cells and enhanced proliferation of mouse fibroblast cells.** (A) NIH 3T3 cells form foci in the presence of cell-permeant Nanog-TAT. NIH 3T3 cells were cultured with 100 nM Nanog-TAT (Peitz et al., 2014) for 8 days; normal medium served as control. Scale bar: 200 μm. (B) Foci formation is dependent on the Nanog-TAT concentration. NIH 3T3 cells were cultured with different concentrations (c) of Nanog-TAT and numbers of foci per well were determined after 8 days. Data are means±s.e.m. (*n*=3). (C) Time dependency of Nanog-TAT-induced foci formation. NIH 3T3 cells were treated with 50 nM Nanog-TAT for 1–6 days as indicated and then Nanog-TAT was withdrawn. Foci formation was quantified after a total culture time of 6 days. Data are means±s.e.m. (*n*=3). (D) Effect of Nanog-TAT on the proliferation of NIH 3T3 cells. To investigate the effects of Nanog-TAT on the proliferation of NIH 3T3 cells, 7.5×10^4^ NIH3T3 cells were plated in 3.5-cm^2^ cell culture dishes and cultured with the indicated concentration of with Nanog-TAT for 10 days; normal medium served as control. Equal cell numbers were replated on day 3 and 7. Cumulative cell numbers are shown. Data are means±s.e.m. (*n*=3). c, control; N, Nanog-TAT. (E,F) Nanog-TAT induces anchorage-independent growth in soft agar. NIH 3T3 cells grown for 6 days with 100 nM Nanog-TAT or control medium were cultured in soft agar. After 19 days, colonies with a diameter >50 µm and >200 µm were counted. Data are means±s.e.m. (*n*=3). c, control; N, Nanog-TAT. (G) Nanog-TAT-induced foci formation is reversible. NIH 3T3 cells growing as a monolayer in control medium were diluted to give a single-cell suspension and replated in the presence of Nanog-TAT. The resulting NIH 3T3 foci culture was again replated as a single-cell suspension and cultured in the presence or absence of Nanog-TAT. Data of counted foci are means (*n*=3). (H) Inhibition of the growth-promoting effect of Nanog-TAT by PI3K inhibitor LY294002 (LY). NIH 3T3 cells were cultured in medium with or without 50 nM Nanog-TAT containing DMSO or 10 µM LY294002 for 6 days. Equal cell numbers were replated on day 3. Cumulative cell numbers are shown. Data are means±s.e.m. (*n*=3). (I) Foci formation of Nanog-TAT-treated NIH 3T3 cells in the presence or absence of LY294002. Foci were counted after 6 days (mock) and after 9 days (LY294002). Data are means±s.e.m. (*n*=3). c, control; N, Nanog-TAT. (J,K) Primary Oct4-GiP MEFs show enhanced proliferation in the presence of Nanog-TAT. Oct4-GiP MEFs were cultured either in medium containing Nanog-TAT (50–100 nM) or control medium for 106 days. Equal cell numbers were replated after each passage and cumulative cell numbers were determined. (J) 50-day-old fibroblasts are shown in a phase-contrast (PC) image. Scale bar: 200 µm. (K) A representative proliferation analysis growth curve is depicted. (L) Nanog-TAT-induced bypass of cellular senescence is associated with chromosomal stability in Oct4-GiP MEF cells. Metaphase spreads of Nanog-TAT-treated (right) and untreated cells (middle) that had been cultured for a high number of passages (P14) were prepared and chromosomes counted. Untreated cells (left) that had been cultured for a low number of passages (P3) served as a control. The percentage of chromosome numbers per nuclei are given. Quantitative evaluation of the counted metaphases was P3, *n*=37; control P14, *n*=32; Nanog-TAT P14, *n*=71.

We recently reported that a cell-permeant recombinant Nanog protein enhances proliferation and maintains pluripotency of mouse ESCs by inhibiting endodermal specification in the absence of leukemia inhibitory factor (Peitz et al., 2014). Here, we set out to use cell-permeant recombinant Nanog as a gain-of-function paradigm in various cellular models in order to assess the putative function of Nanog in somatic cells. NIH 3T3 cells are derived from the mouse embryo and exhibit a strictly contact-inhibited growth of spindle-shaped cells in culture. Nanog protein transduction into NIH 3T3 cells results in three-dimensional growth and formation of cell foci (Fig. 1A), indicating anchorage-independent growth. Foci formation is strictly dependent on the concentration and duration of exposure of Nanog-TAT (Fig. 1B,C). Maximal numbers of foci were observed upon application of 50 nM Nanog-TAT for 5 days (Fig. 1C). In order to assess whether Nanog gain-of-function alone has an impact on the stem cell transcriptional network in NIH 3T3 cells, we analyzed a set of pluripotency-associated genes by semi-quantitative RT-PCR. This analysis revealed that Nanog protein transduction had no impact on the transcript levels of Oct4, Sox2 and Rex-1 (data not shown). To analyze the growth kinetics of Nanog-transduced NIH 3T3 cells, we determined cumulative cell numbers for 10 days. Nanog-TAT caused a strongly increased proliferation, yielding about four-fold more cells within 10 days as compared to the control (Fig. 1D). To rule out putative pleiotropic effects associated with the direct delivery of recombinant protein, we employed a cell-permeant Nanog control protein lacking the homeodomain, designated ΔNanog-TAT. ΔNanog-TAT failed to enhance proliferation of NIH 3T3 cells as determined by cumulative cell numbers after 10 days of treatment, as well as by quantification of foci formation (Fig. S1). To confirm the anchorage-independent growth phenotype induced by full-length Nanog, we assayed the capability of Nanog-TAT-treated cells to grow in soft agar. The number and sizes of the resulting colonies were quantified after 19 days. Nanog protein transduction resulted in growth of more than 250 colonies in a 6-cm dish, 20% of them exceeding a diameter of 200 µm, whereas only few colonies were observed in case of the control (Fig. 1E,F). Both, foci formation and growth in soft agar, indicate contact independent growth due to Nanog activity.

Oncogenes like *ras* are able to stably and irreversibly transform NIH 3T3 cells, and we asked whether the transient intracellular delivery of Nanog also results in stable transformation or represents a transiently occurring phenotype. To address this question, we applied Nanog-TAT for a period of 8 days to NIH 3T3 cells, which led to foci formation. Cells were then passaged and cultured in the presence or absence of Nanog-TAT. The foci formed in the presence of Nanog-TAT were no longer detected after withdrawal of Nanog-TAT, indicating that the transforming effect is a reversible process (Fig. 1G). It has been reported that the overexpression of *Eras* induces a similar oncogenic transformation in somatic cells (Takahashi et al., 2003) involving the phosphatidylinositol 3-kinase (PI3K) cascade, which is known to be important for both transformation (Rodriguez-Viciana et al., 1997) and ESC propagation (Di Cristofano et al., 1998; Sun et al., 1999). Thus, we examined whether PI3K inhibition does interfere with Nanog protein transduction. It turned out that Nanog-TAT is not able to rescue the growth-inhibiting effect of PI3K, suggesting that Nanog depends on PI3K activity (Fig. 1H). In contrast, the transforming property of Nanog-TAT was only slightly affected by PI3K inhibition. The ability to form foci was largely maintained, although foci formation was retarded due to the reduced proliferation of the cells (Fig. 1I). In conclusion, our results demonstrate that Nanog induces loss of contact inhibition through a PI3K-independent mechanism in NIH3T3 cells.

Next, we studied the activity of Nanog protein in murine embryonic fibroblasts (Oct4-GiP MEFs) representing a primary, non-transformed cell population. Nanog transduction induced enhanced proliferation and morphological changes of low passage Oct4-GiP MEFs to a more bipolar shape with an increased nuclear-to-cytoplasmic ratio (Fig. 1J). During long-term culture, control Oct4-GiP MEFs transitionally ceased to proliferate after 4–6 passages, but then resumed expansion, indicative of spontaneous transformation of the cells. Nanog-TAT-treated Oct4-GiP MEFs, in contrast, kept dividing for at least 13 passages (more than 3.5 months) (Fig. 1K). To check the chromosomal integrity, we examined the karyotypes of untreated Oct4-GiP MEF cultures (passage 3) and long-term-cultured cells (passage 14) incubated with or without Nanog-TAT (Fig. 1L). We observed that all metaphases of untreated high-passage cells adopted an aberrant mainly hypo-tetraploid karyotype. Nanog-transduced cells, in contrast, predominantly maintained a normal karyotype, indicating that prolonged expansion of Nanog-TAT-treated cells is not a cause of aneuploidy.

### Nanog suppresses replicative senescence in human primary fibroblasts

**Fig. 2.**
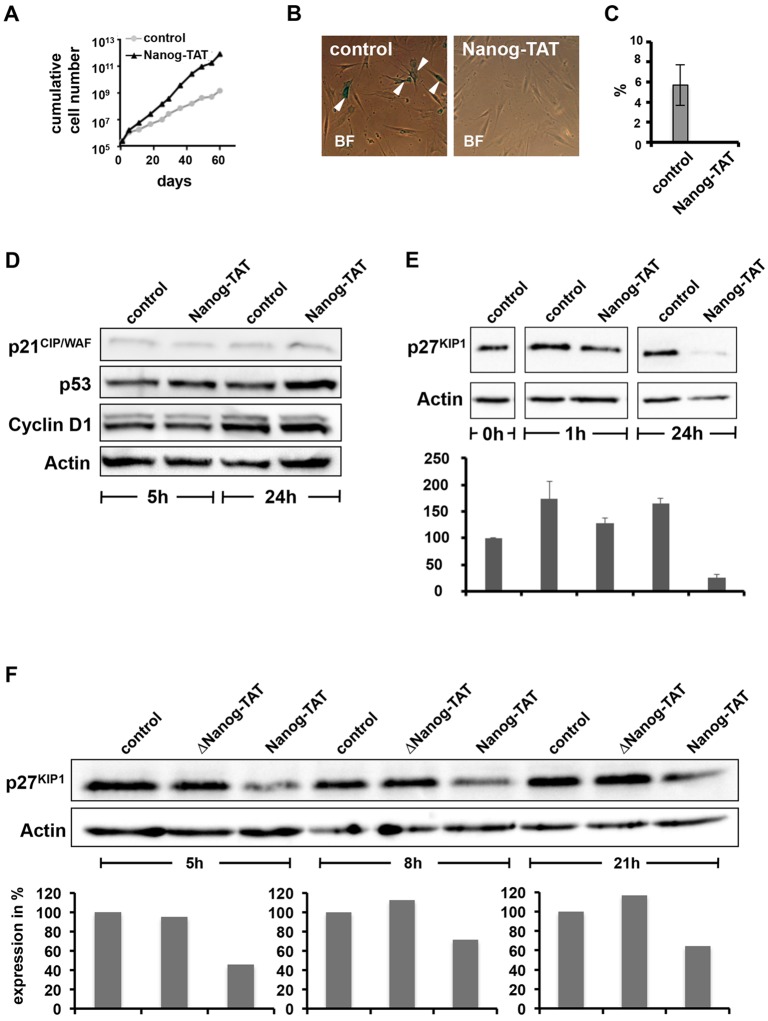
**Nanog suppresses senescence in primary fibroblasts coinciding with low levels of cell cycle kinase inhibitor p27^KIP1^.** (A) Human primary fibroblast cells (MP-hADFs) show enhanced proliferation in the presence of Nanog-TAT. Human fibroblasts were cultured in medium containing 100 nM Nanog-TAT or in control MEF medium. Equal cell numbers were replated after each passage and cumulative cell numbers were determined. A representative proliferation analysis growth curve is depicted. Cumulative cell numbers are shown. (B) A substantial portion of primary human dermal fibroblasts cultured with control medium exhibit senescence-associated SA-β-gal activity (arrowheads), whereas cells cultured with 100 nM Nanog-TAT do not stain for SA-β-gal. BF, bright field. Viewed at a magnification of 20×. (C) A quantification of SA-β-gal-positive cells in the absence or presence of Nanog-TAT is depicted. 5.7% of cells cultivated with control medium are positive for SA-β-gal, whereas no SA-β-gal-positive cells could be observed in the presence of Nanog-TAT. (D,E) The expression levels of different key molecules involved in the cell cycle control were assessed in response to Nanog protein transduction. Oct4-GiP MEFs were synchronized and treated with Nanog-TAT-supplemented medium (100 nM). After 5 h and 24 h, respectively, fibroblasts were harvested and subjected to immunoblot analysis employing the indicated antibodies. Fibroblasts treated with Nanog-TAT show no striking differences in expression of p21^CIP/WAF^, p53 or cyclin D1 compared to Oct4-GiP MEFs treated with control medium. Actin served as a loading control. (E) Oct4-GiP MEFs were synchronized and incubated with control medium or 100 nM Nanog-TAT. After distinct time points, Oct4-GiP MEFs were harvested and subjected to immunoblot analysis with anti-p27^KIP1^ antibody. A representative immunoblot is shown (top panel) showing that p27^KIP1^ is consistently downregulated upon Nanog-TAT treatment. Actin served as a loading control. A mean±s.d. densitometric analysis of p27^KIP1^ expression levels is presented (*n*=3) (bottom panel). (F) Oct4-GiP MEFs were synchronized and incubated with medium only (control), medium containing 50 nM control protein (ΔNanog-TAT) and medium containing Nanog-TAT for the indicated periods of time. Upon culture of Oct4-GiP MEFs with Nanog-TAT, p27^KIP1^ expression is downregulated after 5 h, 8 h and 21 h. Culture of Oct4-GiP MEFs with ΔNanog-TAT does not change protein expression levels of p27^KIP1^ compared to cells treated with control medium (top). The immunoblot was quantified densitometrically and quantification of p27^KIP1^expression is depicted (*n*=2) (bottom). After 5 h of Nanog-TAT treatment the expression of p27^KIP1^ is decreased to ∼45%, after 8 h p27^KIP1^ expression is reduced to ∼70% and after 21 h of Nanog-TAT culture p27^KIP1^ expression is diminished to ∼65%.

Next, we investigated to what extent Nanog has the same effect on primary human cells. With human primary adult dermal fibroblasts (MP-hADFs), we observed an increased proliferation rate after Nanog transduction, which mirrors the effect observed in MEFs. Nanog-TAT-treated cells grew in a densely packed manner, adopted more spindle-like shapes and showed a reduced ratio of cytoplasm to nucleus. From a starting cell number of 250,000 cells, Nanog-TAT-treated fibroblasts exhibited a final cumulative cell number of 8×10^11^ after 10 passages. In contrast, 250,000 MP-hADF fibroblast cells cultured with control medium only gave rise to 1.5×10^9^ cells after 10 passages (Fig. 2A). We reasoned that the capability to enhance proliferation over extended passages might be due to Nanog-induced suppression of replicative senescence. In order to analyze senescence in Nanog-transduced cells, we determined senescence-associated β-galactosidase (SA-β-gal) activity as a means to quantify the number of senescent cells in culture (Dimri et al., 1995). Approximately 6% of MP-hADFs cultured under normal conditions for 3 passages stained positive for SA-β-gal (Fig. 2B,C). In contrast, no SA-β-gal activity was detectable in MP-hADFs cultured in the presence of Nanog-TAT (Fig. 2B,C). These data demonstrate that Nanog activity is able to suppress senescence in primary fibroblast cells.

### Culture of MEFs with Nanog-TAT decreases p27^KIP1^ expression

In order to investigate the senescence-blocking effect induced by Nanog at the molecular level, we analyzed the expression of cell cycle factors. For this, Oct4-GiP MEFs were cultured in low-serum conditions for G0 phase synchronization and treated thereafter with aphidicolin in order to synchronize the cell population in S phase of the cell cycle. Oct4-GiP MEFs were cultured with control medium and Nanog-TAT, respectively. After 5, 8, and 21 h cells were harvested and subjected to semi-quantitative RT-PCR analysis. We analyzed a set of cell cycle factors including p53 (also known as TP53), p16^INK4a^ (also known as CDKN2A), p21^CIP/WAF^ (also known as CDKN1A) and p27^KIP1^. p53 is involved in DNA repair, as well as initiation of apoptosis, in case DNA damage is irreparable. p16^INK4a^ is one marker that can be used for the identification of a senescent phenotype in cells. p21^CIP/WAF^ and p27^KIP1^ are proteins of the Cip/Kip family that act as key cell cycle regulators by inhibiting Cdk2. Besides this, p21^CIP/WAF^ acts downstream of p53 in regulating transition through the cell cycle in G1 phase (el-Deiry et al., 1994). FGF receptor expression can indicate for a proliferative status in fibroblast cells. Semi-quantitative RT-PCR analysis demonstrated that Nanog protein transduction did not yield a significant modulation of the analyzed cell cycle factors except a modest downregulation of p27^KIP1^ (Fig. S2A). After 5 h of Nanog-TAT treatment, the RNA expression level of p27^KIP1^ was slightly reduced, to ∼90% compared to fibroblasts cultured in control medium. After 8 h of Nanog-TAT application, the expression of p27^KIP1^ was diminished to ∼70%. After 21 h of Nanog transduction, we detected only ∼25% p27^KIP1^ transcript as compared to cells incubated with control medium (Fig. S2A). To further determine the p27^KIP1^ modulation quantitative real-time PCR (RT-qPCR) analysis was performed. MEFs were synchronized in the S phase of the cell cycle and cultured with control medium and 100 nM Nanog-TAT for 2.5, 5 and 10 h. Cells were then harvested and subjected to RT-qPCR analysis. We observed gradual downregulation of p27^KIP1^ mRNA upon Nanog-TAT treatment (Fig. S2B). In order to further assess cell cycle factor modulation also at the protein level, cell lysates were prepared after 5 and 24 h of Nanog transduction and subjected to immunoblot analysis. Immunoblotting analysis confirmed that, out of the factors analyzed, p27^KIP1^ is consistently expressed at lower levels after both 5 and 24 h of Nanog transduction (Fig. 2D,E). Quantification demonstrates that 5 h of Nanog transduction is sufficient to reduce the p27^KIP1^ protein level to about half compared to control; 24 h of incubation with Nanog-TAT yielded a further decline of p27^KIP1^ to ∼40% compared to control (Fig. 2E). In contrast, Nanog transduction has no apparent effect on p21^CIP/WAF^, p53 and cyclin D1 levels. To further assess the specificity of this effect, we employed the ΔNanog-TAT protein as a control; ΔNanog-TAT did not result in downregulation of p27^KIP1^ expression at any time point investigated (Fig. 2F).

### ChIP analysis reveals Nanog-binding sites within the p27^KIP1^ locus

**Fig. 3.**
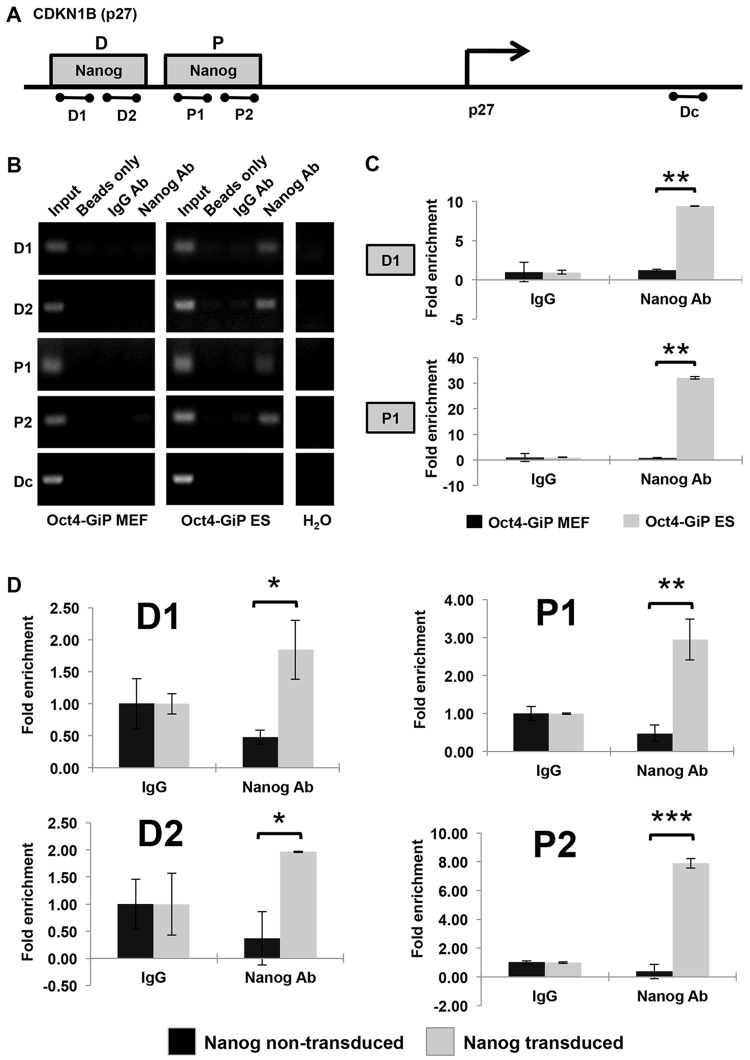
**ChIP analysis reveals Nanog-binding sites regulating p27^KIP1^ expression.** (A) Schematic representation (not drawn to scale) of the p27 genomic locus (RefSeq: NM_009875) highlighting the location of putative Nanog-binding sites designated ‘D’ (primer pairs designated as D1 and D2) and ‘P’ (primer pairs designated as P1 and P2) in the upstream region of the p27^KIP1^ gene (Chen et al., 2008; Marson et al., 2008). PCR primer pairs were designed for these sites for ChIP analyses (dumbbell shaped; Table S2). (B) ChIP analysis reveals that Nanog protein in ESCs binds within the upstream region of the p27^KIP1^ gene. Oct4-GiP MEF and Oct4-GiP ESCs were cultured, harvested and processed for ChIP analysis with beads only, IgG and Nanog antibody. The Oct4-GiP MEF cell line was used as a negative control. Input DNA (10%) was used as a control for ChIP. Beads only and IgG served as negative controls. Putative Nanog-binding regions were amplified by the designed primer pairs (D1, D2, P1 and P2). Primer pairs were also designed randomly in the 3′UTR region of the p27^KIP1^ gene to serve as a negative (desert) control (Dc). (C) RT-qPCR analysis on the ChIP samples explained in B using primers pairs ‘P’ (P1) and ‘D’ (D1) to amplify the Nanog-binding p27^KIP1^ sites. RT-qPCR was also performed on the Dc primer set but no amplification was observed other than the input samples (data not shown). Two independent biological replicates were performed for ChIP analysis. **P<0.01 (two-tailed *t*-test). (D) RT-qPCR analysis on the ChIP samples explained in B derived from MEFs transduced with 100 nM Nanog-TAT. Non-transduced fibroblasts kept in standard medium served as controls. Oct4-GiP MEFs (passage 3) were treated with dialysis buffer only and with 100 nM Nanog-TAT for 10 h, harvested and processed for ChIP analysis with IgG and Nanog antibody. Cells were washed with heparin to remove extracellularly bound Nanog-TAT protein before harvesting fibroblasts. Input DNA (10%) was used as a control for the ChIP. IgG served as negative control. Putative Nanog-binding regions upstream to p27 transcriptional start site were amplified by primer pairs (designated as D1, D2, P1 and P2; Table S2). Primer pairs were also designed randomly in the 3′UTR region of the p27^KIP1^ gene to serve as a negative (desert) control (Dc) but no amplification was observed other than the input samples (data not shown). Two independent biological replicates were performed for ChIP analysis. *P<0.05; **P<0.01; ***P<0.001 (two-tailed *t*-test).

Thus far, our data indicate that Nanog is able to enhance proliferation and to suppress senescence in somatic cells, along with consistently reducing p27^KIP1^ levels. We further asked whether p27^KIP1^ is a direct transcriptional target of Nanog, thus explaining the observed downregulation of p27^KIP1^. Two studies using high-resolution massive parallel DNA sequencing of chromatin immunoprecipitation (ChIP-seq) have reported that there are two Nanog-binding sites in the upstream region of the p27^KIP1^ gene (Fig. 3A; Chen et al., 2008; Marson et al., 2008). To confirm Nanog binding to these sites, designated ‘D’ (primer pairs designated as D1 and D2) and ‘P’ (primer pairs designated as P1 and P2, see Materials and Methods), we performed ChIP analysis using mouse ESCs. Oct4-GiP MEFs served as a negative control given that they do not express Nanog. ChIP analysis with a Nanog-specific antibody revealed amplification of Nanog-bound regions D and P upstream of the p27^KIP1^ as judged by both semi-quantitative PCR (Fig. 3B) and RT-qPCR analysis (Fig. 3C). Amplification was not observed using the Oct4-GiP MEF control cells, with beads only or with unspecific IgG. Moreover, specificity is confirmed by the observation that a desert control lying within the p27^KIP1^ gene was not amplified (Fig. 3B). This observation confirms that there are Nanog-binding sites in the upstream region of the p27^KIP1^ gene.

To demonstrate the binding of Nanog to these p27^KIP1^ upstream regions in fibroblasts after protein transduction, we applied 100 nM of Nanog-TAT to Oct4-GiP MEFs. Oct4-GiP MEFs treated with vehicle only served as a negative control. ChIP analysis employing a Nanog-specific antibody showed amplification of Nanog-bound regions upstream to the p27^KIP1^ gene only in Oct4-GiP MEF cells transduced with Nanog (Fig. 3D). This data demonstrates that Nanog binds to the upstream region of the p27^KIP1^ gene in Nanog-TAT-transduced fibroblasts, indicating direct regulation of its expression.

## DISCUSSION

In this study, we set out to analyze a putative function of the pluripotency factor Nanog in somatic cells. We employed a cell-permeant version of Nanog as a non-DNA-based and non-genetic paradigm to modulate cellular function. We found that Nanog protein transduction enhanced proliferation of both NIH 3T3 cells and primary mouse and human fibroblasts. Moreover, Nanog induces anchorage-independent growth of NIH 3T3 cells in a dose- and time-dependent manner, which otherwise typically exhibit strict contact inhibition. Previously, it had been reported that genetic ectopic expression of Nanog in NIH 3T3 cells causes an enhanced proliferation (Zhang et al., 2005) and foci formation, as well as growth in soft agar (Piestun et al., 2006). Nanog has also been reported to enhance proliferation and/or self-renewal in other somatic and stem cell lines by regulating molecules involved in stemness, cell cycle and senescence machinery [Cao et al., 2010 (adult human fibroblasts); Choi et al., 2012 (embryonic carcinoma cells); Go et al., 2008 (mesenchymal stem cells); Shan et al., 2012 (cancer stem cells); Tanaka et al., 2007 (hematopoietic stem cells)]. Thus, we conclude that our Nanog gain-of-function paradigm that is achieved by direct protein delivery is functional and induces loss of contact inhibition and gain in proliferation. Moreover, we demonstrate that PI3K inhibition does not substantially affect induction of anchorage-independent growth, whereas Nanog is not able to overcome the growth-inhibiting effect induced by PI3K inhibition. In primary fibroblasts, Nanog protein transduction induces enhanced proliferation, enabling prolonged culture for more than 13 passages seemingly bypassing cellular senescence. Indeed, we show that Nanog transduction into primary human fibroblasts results in effective suppression of SA-β-gal activity, a widely used marker for senescence. The expansion of primary human cells by transient transduction of Nanog protein might be of general interest, in particular for *in vitro* expansion of cells.

Our study shows that Nanog protein transduction in mouse fibroblasts specifically results in low expression of p27^KIP1^ both at the RNA and protein level, indicating a functional link between the pluripotency transcriptional network and the cell cycle machinery. We confirmed two putative Nanog-binding sites in the 5′ upstream activating sequence of the p27^KIP1^ gene (Chen et al., 2008; Marson et al., 2008) by ChIP analyses both in ESCs and Nanog-transduced MEFs. Recently, it has been reported that Oct4 represses p21 expression to contribute to ESC proliferation and self-renewal (Lee et al., 2010). p27^KIP1^ together with p21^CIP/WAF^ represent the major cyclin-dependent kinase inhibitors (CKIs) regulating G1 to S transition. Ablation of p27^KIP1^ in mice results in multiorgan hyperplasia (Fero et al., 1996) and numerous studies *in vitro* confirm a regulatory control of p27^KIP1^ overproliferation. Notably, ESCs do lack the ability to enter into senescence and their growth is not subject to contact inhibition or anchorage dependence. Given our observation that Nanog gain-of-function is able to suppress contact inhibition and senescence while downregulating p27^KIP1^, we provide a functional link between Nanog and cell cycle regulator p27^KIP1^ at the molecular level.

## MATERIALS AND METHODS

### Cell culture

NIH 3T3 MEFs, Oct4-GiP MEFs and primary human adult dermal MP fibroblasts (MP-hADF) were cultured in Dulbecco's modified Eagle medium (DMEM, GIBCO) containing 10% fetal calf serum (FCS; GIBCO), 100 U/ml penicillin and 0.1 mg/ml streptomycin (GIBCO). In all experiments medium was changed daily. Culture of Oct4-GiP ESCs (Oct4-GiP; Ying et al., 2002) for ChIP analysis was performed on 0.1% gelatin-coated dishes (Sigma-Aldrich) in high-glucose DMEM (GIBCO) with 15% FCS, 1% non-essential amino acids (GIBCO), 1 mM sodium pyruvate (GIBCO), 2 mM L-glutamine (GIBCO) and 100 µM β-mercaptoethanol (GIBCO) and 1000 U/ml leukemia inhibitory factor (LIF; Millipore). Differentiated cells were counter-selected from Oct4-GiP ESCs by adding 1 µg/ml puromycin (GIBCO) for at least 1 week before an experiment.

For growth curve analysis of Oct4-GiP MEFs, 20,000 cells at passage number 1 were plated in a 3.5-cm^2^ dish and cultured in the absence and presence of 50–100 nM Nanog-TAT over several passages. As soon as cells reached 80–90% confluency, the cells were sub-cultured and again 20,000 cells were replated. After each passage, cells were counted and the cumulative cell numbers were determined in order to assess the proliferation rate.

For growth curve analysis of MP-hADF cells, 250,000 cells were seeded in a 6-cm dish and cultured in the absence and presence of 100 nM Nanog-TAT over several passages. As soon as cells reached 80–90% confluency, the cells were split and again 250,000 cells were re-seeded. After each passage, cells were counted and the cumulative cell numbers were determined in order to assess the proliferation rate.

For cell proliferation analysis of Oct4-GiP MEFs cultured with Nanog-TAT, ΔNanog-TAT or control medium by RT-PCR or immunoblotting, 750,000 cells at passage number 5 were seeded on a 10-cm cell culture dish. In order to synchronize the fibroblasts, cells underwent serum starvation (i.e. a concentration of 0.2% FCS) for 48 h. This led to an accumulation of cells in G0 phase of the cell cycle. Subsequently Oct4-GiP MEFs were cultured in MEF medium containing 10% FCS, but the medium was supplemented with 4 µg/ml aphidicolin (Sigma-Aldrich) for another 16 h to finally synchronize cells in S phase of the cell cycle. Afterwards, Oct4-GiP MEFs were washed twice with PBS and cultured with 100 nM of Nanog-TAT, 50 nM of ΔNanog-TAT or control medium for the indicated times.

### Plasmid construction and preparation of recombinant fusion proteins

Plasmid generation of the pTriEx1.1 vector harboring the genetic information for Nanog-TAT (NLS-Nanog-TAT-H6) for expression and native purification of recombinant protein is described elsewhere (Peitz et al., 2014). For NLS-ΔNanog-TAT-H6 (without the 60-amino-acid long homeodomain), PCR fragments encompassing the open reading frames for NLS-ΔNanog-TAT-H6 (hereafter ΔNanog-TAT) flanked by NcoI-XhoI sites were synthesized and inserted into the NcoI-XhoI sites of pTriEx1.1 (Novagen, UK). Native purification of ΔNanog-TAT was performed as described elsewhere for Nanog-TAT (Peitz et al., 2014) with some modifications. For bacterial overexpression of ΔNanog-TAT, overnight cultures (LB containing 0.5% glucose and 50 µg/ml carbenicillin) were inoculated with freshly transformed BL21 (DE3) GOLD cells (Stratagene) and cultured at 30°C. Expression cultures (TB containing 0.5% glucose and 100 µg/ml ampicillin) were grown at 37°C and induced to an optical density at 600 nm (OD_600_) of 1.5 with 0.5 mM isopropyl-β-D-thiogalactopyranoside (IPTG) for 1 h. Pellets were resuspended in lysis buffer (2 mM imidazole, 500 mM NaCl, 50 mM Na_2_HPO_4_, 5 mM Tris-HCl, pH 7.8), and lysozyme (Sigma-Aldrich) and benzonase (Novagen, UK) was sequentially added, each for 20 min at 4°C. After a centrifugation step (20,000 ***g*** for 30 min), the supernatant was incubated for 1 h with 1 ml Ni-NTA slurry (Qiagen, Hilden, Germany) per liter initial culture. The resin was packed in a gravity column, washed (100 mM imidazole, 500 mM NaCl, 50 mM Na_2_HPO_4_, 5 mM Tris-HCl, pH 7.8) with 6 bed volumes (the volume of beads after sedimentation of slurry by gravity) and eluted (250 mM imidazole, 500 mM NaCl, 50 mM Na_2_HPO_4_, 5 mM Tris, pH 7.8) with 8 bed volumes. Eluted fractions were successively dialyzed against PBS followed by non-supplemented KnockOut-DMEM (GIBCO).

### Nanog protein transduction

Medium for transduction experiments was prepared by mixing Nanog dialysate fractions in a ratio of 1:1 with double supplemented medium (AdvDMEM; GIBCO) additionally supplemented with 2% FCS, 1% insulin-transferrin-selenium solution (ITS; GIBCO), 1% non-essential amino acids, 4 mM glutamine and 200 µM β-mercaptoethanol. The mixture was incubated in a water bath for 2 h at 37°C and precipitations were cleared by centrifugation (6,000 ***g*** for 5 min) and sterile filtration. FCS was then added to a final concentration of 5%. Final Nanog-TAT concentration in FCS-containing medium was determined by dot blot analysis with Nanog-TAT dialysis fraction serving as standard.

### Foci formation and soft agar assay

NIH 3T3 cells were grown in the presence of Nanog-TAT in 12-well plates and seeded at a low density (10^3^ cells/well). Untreated cells served as a control. Medium was changed every day. After 8 to 10 days the number of three-dimensional foci was counted. For soft agar assays, 6-cm petri-dishes were covered with 5 ml of the appropriate medium containing 0.5% agarose. NIH 3T3 cells were pre-incubated with or without Nanog-TAT for 6 days. 10^4^ cells were suspended in 2 ml of the appropriate medium containing 0.3% agarose and with or without Nanog-TAT (25–100 nM), and added to each plate. We added 1 ml of control medium or medium containing Nanog-TAT weekly, respectively. After 19 days of growth in soft agar, the diameters of colonies were measured and numbers of colonies were counted.

### SA-β-gal staining

For the analysis of SA-β-gal expression, MP-hADFs at passage 16, already cultured with 100 nM of Nanog-TAT for 2 weeks were seeded at a density of 250,000 cells per 6-cm dish. The next day, cells were fixed and stained for SA-β-gal expression (Dimri et al., 1995).

### Karyotype analysis

80% confluent Oct4-GiP MEFs were incubated with 0.1 µg/ml colcemid (Gibco^®^ KaryoMAX^®^ Colcemid™ Solution in HBSS) for 16 h. Cells were then trypsinized and resuspended in hypotonic KCl solution (0.075 M), incubated for 10 min at room temperature and fixed with methanol and glacial acetic acid (3:1). Chromosomes were visualized using Giemsa dye.

### Western blotting

For SDS-PAGE analysis, gels exhibiting a percentage of 10% or 15% (bis)acrylamide were used. SDS-PAGE-separated protein samples were blotted onto a nitrocellulose membrane employing the wet blot technique. Blotting was performed for 1 h at 100 V. For cell cycle analysis, we employed the following antibodies against p21^CIP/WAF^ (556431; mouse IgG, 1:200; BD Pharmingen, Heidelberg, Germany), p27^KIP1^ (554069; mouse IgG, 1:200; BD Pharmingen, Heidelberg, Germany), p53 (1C12; mouse IgG, 1:2000; Cell Signaling, Frankfurt, Germany) and cyclin D1 (DCS-6; mouse IgG, 1:200; BD Pharmingen, Heidelberg, Germany). Cyclin D1 exhibits an additional band in immunoblot analysis. This phenomenon observed is due to the rodent origin of cells, as stated in the datasheet provided by the manufacturer of the antibody. As secondary antibody, we utilized horseradish peroxidase (HRP)-linked anti-mouse IgG (7076; 1:200–1:1000; Cell Signaling Technology, Frankfurt, Germany) antibody. Detection was carried out with SuperSignal West Pico Chemiluminescent Substrate (PIERCE) or Supersignal West Femto Chemiluminescent Substrate (PIERCE), respectively.

### RT-PCR

RNA from aggregates or somatic cells was isolated with the SV Total RNA Isolation System (Promega), RNeasy Mini Kit (Qiagen Inc) or Trizol (Invitrogen), and reverse-transcribed with M-MLV Reverse Transcriptase, RNase H Minus, Point Mutant (Promega) or iScript reverse transcriptase (Bio-Rad). PCR reactions were performed using GoTaq (Promega). Primers used for RT-PCR are listed in Table S1.

### Chromatin immunoprecipitation

For ChIP analysis, Oct4-GiP MEFs, Oct4-GiP ESCs were used. Proteins bound to DNA were cross-linked using 1% formaldehyde for 7 min at room temperature followed by quenching the fixation reaction by addition of glycine (0.125 M final concentration) for 5 min at room temperature. After washing with ice-cold PBS, cells were collected by centrifugation and were lysed in lysis buffer [50 mM Tris-HCl pH 8.1, 10 mM EDTA, 0.1% SDS and Complete protease inhibitors (Roche)] for 10 min on ice and further sonicated using Bioruptor (Diagenode) and centrifuged twice (20,000 ***g*** for 15 min), and the supernatant was frozen at −80°C. The supernatant was thawed and 50 µg of chromatin was diluted 10 times in dilution buffer (16.7 mM Tris-HCl pH 8.0, 167 mM NaCl, 0.01% SDS, 1.1% Triton X-100, 1.2 mM EDTA and Complete protease inhibitors). 5 µg (10%) of input was taken as a control. After dilution, the protein–DNA complexes were immunoprecipitated overnight at 4°C with rotation using primary antibodies against Nanog (AB5731, Millipore) and rabbit control IgG ChIP grade (ab46540, Abcam). Beads only were also taken as a negative control. Immunoprecipitated chromatin was incubated with Protein A/G Plus agarose beads (sc-2003; Santa Cruz Biotechnology) for another 1 h at 4°C and then washed three times with low-salt washing buffer (20 mM Tris-HCl pH 8.0, 1% Triton X-100, 0.1% SDS, 150 mM NaCl, 2 mM EDTA and 0.01% Tween 20) and twice with high-salt washing buffer (20 mM Tris-HCl pH 8.0, 1% Triton X-100, 0.1% SDS, 500 mM NaCl, 2 mM EDTA and 0.01% Tween 20). The protein–DNA complexes bound to the beads were eluted by incubation with elution buffer (1% SDS, 0.1 M NaHCO_3_) and subsequently treated with RNAse (2 h; Concert™, Carlsbad) and Proteinase K (2 h; Roche, Mannheim, Germany). Cross-linking was then reversed by incubation overnight at 65°C. Bound DNA was purified by Wizard SV Gel and PCR clean-up kit (Promega) and the eluted DNA was then used for both semi-quantitative and RT-qPCR for analysis. RT-qPCR was performed using the SYBR-green Supermix (Bio-Rad, Hercules, CA, USA) on an Eppendorf realplex Mastercycler and the data was analyzed using the fold enrichment method. Primers used for both semi-quantitative PCR and RT-qPCR to identify the putative Nanog-binding sites in the p27^KIP1^ genomic region regulating the p27^KIP1^ expression and the desert control are listed in Table S2. Two biological replicates were performed for this experiment. ChIP analysis following the above protocol was also performed on Nanog-TAT-transduced and non-transduced Oct4-GiP MEFs (passage 3). Cells were washed with heparin (0.5 mg/ml in PBS; Sigma-Aldrich) to remove non-internalized protein before harvesting cells.

### Statistical analysis

Statistical analysis to calculate *P*-value was carried out using a two-tailed test. The level of significance was set to *P*<0.05.
